# Quantitative Assessment of Head Tremor in Patients with Essential Tremor and Cervical Dystonia by Using Inertial Sensors

**DOI:** 10.3390/s19194246

**Published:** 2019-09-30

**Authors:** Lazar Berbakov, Čarna Jovanović, Marina Svetel, Jelena Vasiljević, Goran Dimić, Nenad Radulović

**Affiliations:** 1 Institute Mihailo Pupin, University of Belgrade, Volgina 15, 11050 Belgrade, Serbia; jelena.vasiljevic@pupin.rs (J.V.); goran.dimic@pupin.rs (G.D.); 2 Clinic of Neurology, School of Medicine, University of Belgrade, 11000 Belgrade, Serbia; carnajov@gmail.com (Č.J.); marinasvetel@gmail.com (M.S.); 3 Math Modeling, 11000 Belgrade, Serbia; nradulovic@mathmodeling.rs

**Keywords:** neurological tremor, ambient assisted living, remote healthcare, essential tremor, cervical dystonia

## Abstract

Tremor is most common among the movement disabilities that affect older people, having a prevalence rate of 4.6% in the population older than 65 years. Despite this, distinguishing different types of tremors is clinically challenging, often leading to misdiagnosis. However, due to advances in microelectronics and wireless communication, it is now possible to easily monitor tremor in hospitals and even in home environments. In this paper, we propose an architecture of a system for remote health-care and one possible implementation of such system focused on head tremor monitoring. In particular, the aim of the study presented here was to test new tools for differentiating essential tremor from dystonic tremor. To that aim, we propose a number of temporal and spectral features that are calculated from measured gyroscope signals, and identify those that provide optimal differentiation between two groups. The mean signal amplitude feature results in sensitivity = 0.8537 and specificity = 0.8039 in distinguishing patients having cervical dystonia with or without tremor. In addition, mean signal amplitude was shown to be significantly higher in patients with essential tremor than in patients with cervical dystonia, whereas the mean peak frequency is not different between two groups.

## 1. Introduction

In recent years, most of the countries in the world have experienced the rise in life expectancy and the increase in aging population. This trend, which initially emerged in developed countries, can now be observed in almost all developing countries. According to the report on World Population Prospects: the 2019 Revision [1], in 2018, for the first time, the number of persons older than 65 years was greater than the number of children younger than 5 years. In addition, it is estimated that, by the year 2050, 16% of the world’s population will be over age 65, in comparison to 9% in 2019. These trends have significantly contributed to increased incidence rate of disabilities and chronic diseases which put additional demands on the health care systems [2]. One of the possible solutions to deal with this challenge, which could enable elderly and disabled people to live independently for as long as possible in their own homes, is to apply Ambient Assisted Living (AAL) concepts within long-term health care support.

AAL is a relatively new approach where the goal is to deploy a number of intelligent devices into the home environment with the aim of supporting elderly or disabled people without intrusive monitoring of their daily activities. One of the main pillars in supporting this new way of providing remote health care is Internet of Things (IoT), which enables seamless collection of data, local processing and control actions. Although IoT cannot completely replace human care-givers, it can undoubtedly improve the patient’s quality of life on a day-to-day basis, enhance their safety and allow them to stay in a comfortable environment for as long as possible, without putting a large financial burden on the already fragile health care systems.

Among the movement disabilities that usually affect older people, tremor is the most common having a prevalence rate of 4.6% in the population older than 65 years [3] and an incidence rate of 616 per 100,000 person-years [4]. Tremor is defined as an involuntary, rhythmic, oscillatory movement of a body part [5]. Despite being one of the most common neurological disorders, distinguishing different types of tremor is sometimes clinically challenging leading to misdiagnosis. Some studies even suggest that one in three patients with tremor was misdiagnosed as having essential tremor, which was the most frequent in patients with dystonia or Parkinson’s disease [6].

Although the tremor itself is not considered a life-threatening condition, it usually generates social anxiety, making everyday activities such as eating, writing, and reading very challenging for the affected population. Tremor is usually treated in a hospital environment, where the patients periodically visit the clinicians who check their symptoms and adjust the therapy as needed. The tremor symptoms may be emphasized due to anxiety experienced during medical checks. Nevertheless, this still has to be confirmed in the future study which will include measurements from both hospital and home environments. A possibility to monitor tremor symptoms in home environments more frequently would provide medical practitioners with valuable additional data, possibly leading to a different and more personalized approach to treating one’s medical condition. This approach can be easily applied nowadays due to recent advances in Micro-ElectroMechanical Systems (MEMS) technologies, low power computation and wireless communications. These technologies allow low-cost monitoring of patient’s condition and in such a way enable objective assessment of patient status in real-time, paving the way for application of Machine Learning (ML) algorithms and Artificial Intelligence (AI) based on such a large amount of collected data.

Tremor monitoring by using inertial sensors is not new, and it has already been considered in a number of research projects for different types of neurological disorders. In the work [7], the authors provide a review of state-of-the-art methods for assessment of neurological tremors in humans and compare the pros and cons of different types of sensors. Real-time estimation of tremor parameters from Gyroscope signals is presented in the work of Gallego et al. [8], where the authors propose an algorithm that extracts the patterns of the tremor from voluntary movements and estimates its instantaneous amplitude and frequency. On the other hand, filtering of tremor signals acquired by accelerometers for real-time applications is presented in [9], where the authors propose two improved algorithms by considering that the tremor contains multiple dominant frequencies over the entire signal duration. The possibility to use inertial sensors for monitoring of the effect of medication on Parkinson’s disease patients has been presented in the work of Teskey et al. [10]. In that work, the authors show that the motion due to Parkinson Disease, when a patient is medicated, does still differ significantly from control motion to allow researchers to quantify potential deficiencies due to use of medication.

A complete end-to-end Personal health systems for monitoring and point-of-care diagnostics (PERFORM) of Parkinson’s disease patients is described in the work of Tzallas et al. [11]. PERFORM system is based on different wearable sensors which constantly monitor a number of motor signals of the patients and pre-process them. In such a way, it allows medical practitioners to remotely monitor the status of the patients, adjust medication doses and provide more personalized treatment. A possibility to employ already available inertial sensors embedded in common smart phones has been considered in the work of Kostikis et al. [12], where the authors proposed machine learning techniques capable of correctly classifying 90% of healthy subjects and 83% of the patients. Another study conducted by using an ambulatory system for quantification of tremor and bradykinesia in patients with Parkinson’s disease was presented in the work of Salarian et al. [13]. In that work, the authors propose an algorithm for tremor detection that showed an overall sensitivity of 99.5% where the estimated tremor amplitude showed a high correlation to the Unified Parkinson’s Disease Rating Scale (UPDRS). A similar study was presented in the work [14], where the authors employed hidden Markov models to estimate the tremor type (resting, action and postural) and its severity. An algorithm capable of characterizing finger and wrist tremor is proposed in the work of Zhou et al. [15], where the results show that Parkinsonian tremor produce oscillations of the hand with different harmonics. In the work [16], the authors considered tremor signals acquired by the accelerometer and developed least-square estimation models to assess the severity of Parkinson’s disease tremor, which was then verified by comparing it with an electromagnetic motion system. Similarly to [16], in a research paper by Jeon et al. [17], the authors compare different machine learning techniques in order to predict the UPDRS, to assist neurologists in disease assessment. In their work, a decision tree was identified as the best classifier with the highest accuracy of 85.55%.

Besides focusing on one specific disease only (e.g., only patients with Parkinson’s disease), there exists a research project that conducted analysis of different disorders with the possibility to differentiate among them by employing intelligent algorithms. In the work [18], the authors propose a new metric capable of differentiating between Parkinson’s and essential tremor symptoms by employing measurements from triaxial accelerometers. The tremor stability index—TSI—proposed therein and calculated by considering the instantaneous frequency variation is shown to have good classification accuracy due to the fact that it describes the difference between two types of tremors well. In a similar manner, the authors in the work of Shaikh et al. [19] consider groups of patients with cervical dystonia and essential tremor and quantify the limb tremor. By analyzing the signals recorded with triaxial accelerometer, they found that the limb tremor amplitude was significantly higher in patients with essential tremor than in patients with cervical dystonia.

### 1.1. Contributions

In this paper, we present the architecture of the system for remote monitoring of patients, which can be applied in a broader context of AAL, along with one possible implementation of such system focused on patients with movement disorders. In particular, here the focus is on two groups: patients with essential tremor and patients with cervical dystonia accompanied by tremor. The aim of the study presented in this paper is to test new tools for differentiating essential tremor from the dystonic one. The tremor signals were acquired by placing the device with embedded inertial sensors on the patient’s head in the hospital during a periodical medical check, and the analysis of the collected data was performed by using Matlab software (version R2018b). The contributions by this paper are as follows:

1. We focus on the head tremor, in contrast to [19], where limb tremor is analyzed, and consider a number of features for differentiation of different types of tremors.
2. An analysis of triaxial gyroscope signals for patients with cervical dystonia with and without head tremor.
3. An analysis of triaxial gyroscope signals for patients diagnosed with essential tremor and cervical dystonia with head tremor.

## 2. Materials and Methods

### 2.1. Subjects

This study included 171 patients with cervical dystonia and 78 patients with essential tremor, who have been asked to sit comfortably in the chair during data collection. The examination and data collection was carried out according to the ethical standards of the Declaration of Helsinki, and approved by the Ethical Committee of the School of Medicine, University of Belgrade. In addition, all of the participants in the study have provided informed consent prior to their participation. This study focuses on following analysis:

1. Group A1: Cervical dystonia without tremor vs. Group B1: Cervical dystonia with tremor present either on head or both head and hands
2. Group A2: Essential tremor on head vs. Group B2: Cervical dystonia with tremor present either on head or both head and hands

In Table 1 and Table 2, we provide clinical and demographic characteristics of the patients belonging to groups A1; B1 and A2; B2 respectively.

### 2.2. Data Acquisition

**Figure 1:**
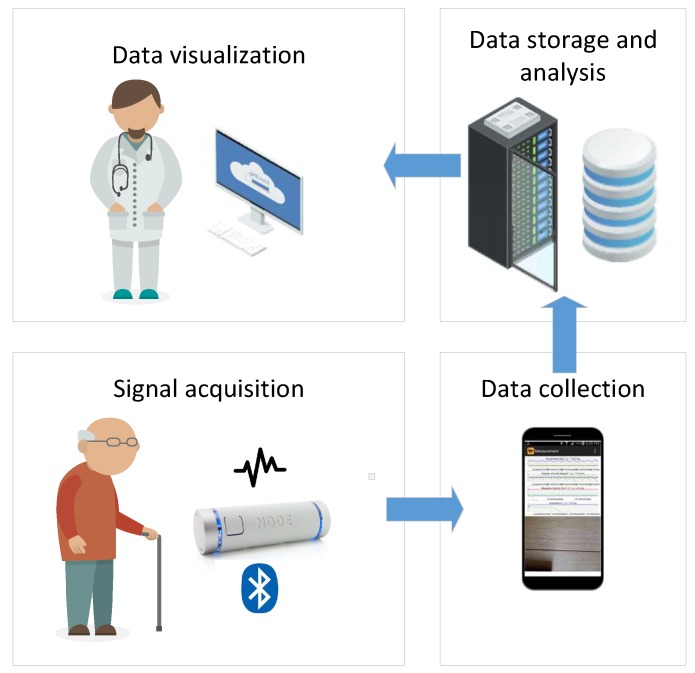
Architecture of Ambient Assisted Living system for remote patient monitoring.

In Figure 1, we present the architecture of the system for acquisition of tremor signal in the context of Ambient Assisted Living, although it can be applied for acquisition for other types of bio-medical and environmental data. As can be seen, the architecture consists of four main building blocks:

- Signal acquisition: which aims to collect bio-medical and other relevant environmental data and send them to the subsequent data collection block. It consists of one or more sensors (biomedical sensors, occupancy and movement sensors, etc.) that are usually connected wirelessly with the rest of the system.
- Data collection: whose goal is to collect the measurements from different sensors. In our study, an Android smart phone device performs this task, since it has enough computational capabilities and storage, while also supporting different network technologies (3G, 4G, WiFi) that enable seamless connectivity towards the internet and the cloud server where the data are stored and analyzed. In addition, it is possible to implement this unit on an inexpensive computer board serving as an edge node (e.g., Raspberry Pi 4) by using e.g., AndroidThings operating system as the development platform.
- Data storage and analysis block configured as a cloud server. The purpose of this block is to get data from different data collection units deployed in patients’ homes, their storage in the local database and analysis of raw data by using different signal processing and ML algorithms.
- Data visualization implemented as a desktop or web application which enables access to the raw and processed data stored in the data storage and analysis block.

**Figure 2:**
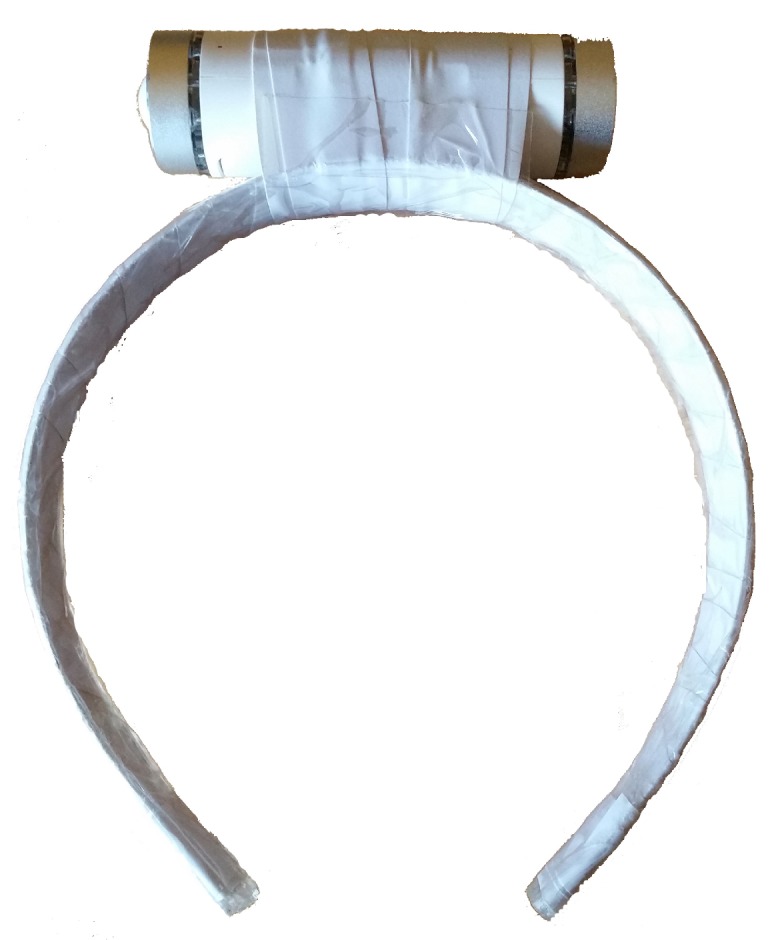
Node + sensor platform setup for head tremor assessment.

**Figure 3:**
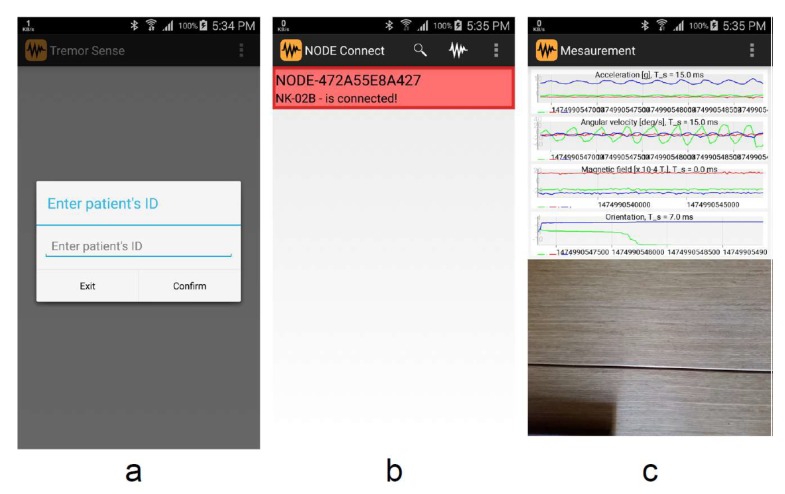
TremorSense Android mobile application.

In this study, for the sake of prototyping of proof of concept and identifying suitable data processing algorithms, we implemented the aforementioned architectural blocks in the following manner:

- Signal acquisition: In this study, we employed Node+ sensor platform to measure tremor signals [20]. Node+ consists of a Bluetooth enabled main board with 3 different sensors: accelerometer, gyroscope and magnetometer that are able to measure the corresponding physical phenomena in 3 dimensions. Node+ is capable of real-time streaming of measurements with sampling frequency up to 70 Hz. The accelerometer can measure up to ±2, 4, 8 and 16 g (with resolution 61 μg, 122 μg, 244 μg, and 488 μg) along all three axes, whereas the gyroscope has angular velocity range of up to ±250, 500, 1000 and 2000 degrees per second (with resolution 0.00763, 0.015, 0.03 and 0.06 degrees per second). Finally, magnetometer’s range is ±1200 μT. For the purpose of measuring tremor in the patient’s head, Node+ is attached to the support as it has been shown in Figure 2.
- Data collection software are implemented as a standalone Android application TremorSense, which is capable of connecting to Node+ sensor platform via Bluetooth, as it is shown in Figure 3. Once TremorSense application is started, a medical practitioner sets the Patient ID number (see Figure 3a). In the next step, she is provided with the list of nearby Bluetooth devices, and prompted to choose the one used for data acquisition (as shown in Figure 3b). Finally, once the Node+ device is connected, a signal acquisition starts and the raw data measurements are shown on the screen, as can be seen in Figure 3c. In the future version of the mobile app, our aim is to perform the signal analysis in the app itself, so that the user can get instant feedback about the results of analysis. In the context of AAL, a user would be the patient itself, and she would be allowed to log into the application with previously provided credentials.
- Data storage and analysis block are implemented as a web application capable of storing uploaded measured data (in .csv format) in the local server file system, and allowing an authorized user to download them to its PC computer. Currently, the data analysis is performed by using Matlab software. The aim is to further improve the web application with analysis capabilities deployed on the server, once the optimal signal processing algorithms have been identified.
- Data visualization is enabled by using Matlab visualization functions. The future work will involve development of standalone desktop or web application, which will be customized according to the requirements provided by medical practitioners.

### 2.3. Data Analysis

**Figure 4:**
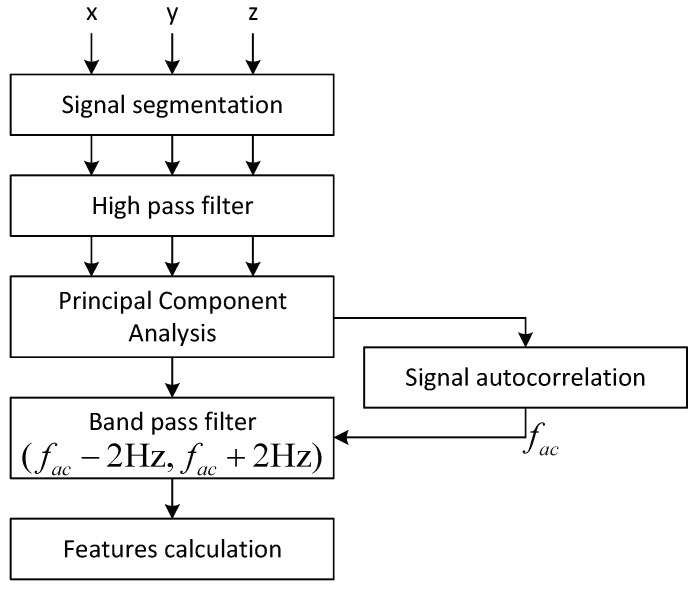
Tremor signal processing chain.

**Figure 5:**
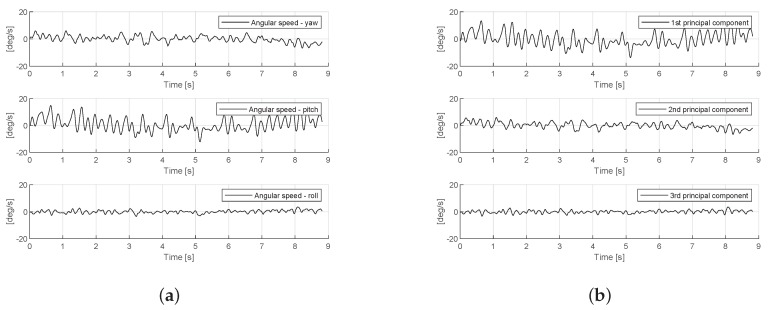
Gyroscope signal measured at the head of a patient with cervical dystonia (Patient-48). (**a**) measured signal from triaxial gyroscope; (**b**) principal components of the measured signal.

**Figure 6:**
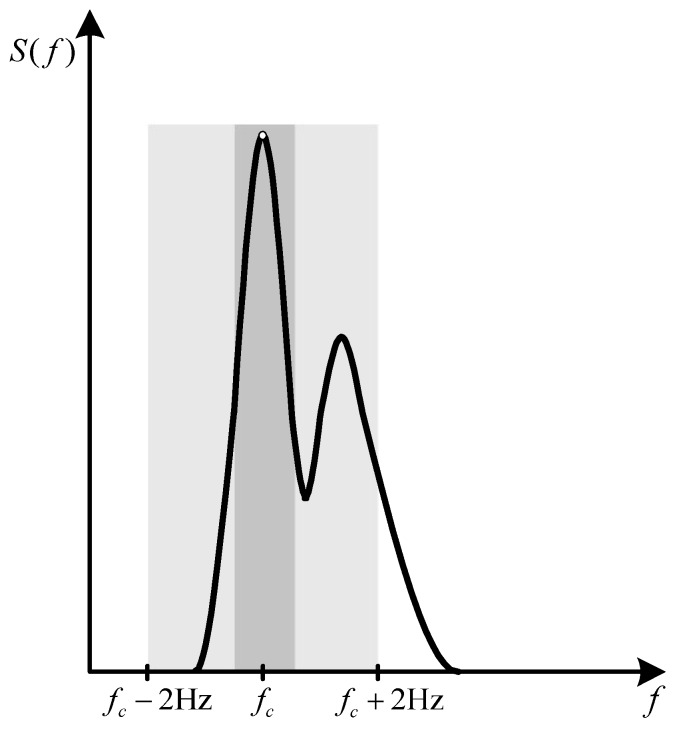
Spectrum of the tremor signal measured by a triaxial gyroscope.

**Figure 7:**
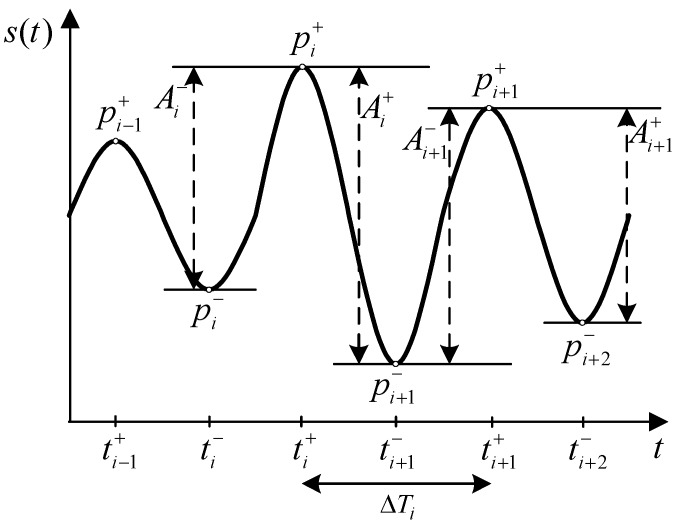
Temporal features of the tremor signal.

All the signals from the triaxial gyroscope are recorded with the sample period of 15 ms that corresponds to a 66.67 Hz sampling rate for a total duration of 10 s. The bandwidth of the gyroscope is 30 Hz, which covers the expected frequencies of the head tremor. Such recording duration was chosen in order not to make patients remain in uncomfortable positions for a long time. The tester is provided with the live measurement shown on the mobile application screen, so that she can track in real-time whether the measurements are correct, and no other type of motion, which could potentially ruin the measured signal, is accidentally made by the patient. The measurements are repeated until the recording of good quality is obtained. The signals are streamed via Bluetooth connection to a TremorSense Android application that is installed on a smart phone, where the data are stored in a .csv file with 4 columns (timestamp, *x*-axis, *y*-axis and *z*-axis measurement). Once the particular recording has been created, it is uploaded to the remote server, so that it can be downloaded for analysis by using Matlab software. As it can be seen in the signal processing chain, which is presented in Figure 4, firstly the signal is segmented by cutting the first and the last 10% of the overall duration in order to focus only on the stable part of the signal. Next, the signal is filtered by using a high-pass Butterworth IIR filter with zero phase and cutoff frequency of 0.2 Hz, in order to correct the trend that cannot be attributed to the tremor. Then, principal component analysis (PCA) is performed on the 3D signal obtained from the gyroscope (The reason why, in this work, we considered the gyroscope measurement is because the head tremor is rotational motion, which can be properly sensed by a gyroscope, free of gravitational effect (like in the case of accelerometer), as it is shown in [21].) sensor in order to isolate the dominant axis of the tremor, similarly to [18]. As it is shown in Figure 5, PCA can be seen as a physical rotation of the sensor, so that the greatest contribution to tremors can be attributed to the first principal component. In the sequel, we focus exclusively on a 1D signal which represents the first principal component. The next step includes the calculation of auto-correlation of the signal that is used in order to calculate the dominant frequency of the signal $f_{ac}$. Next, the signal is passed through a band-pass filter composed of separate low-pass and high-pass filter with cut-off frequency set to $f_{ac}-2$ Hz and $f_{ac}+2$ Hz, respectively. Finally, this signal is used as a basis for calculation of a number of features as follows:

- Peak frequency of the Fast Fourier Transform (FFT) spectrum $f_{c}$ which is calculated by identifying the maximum peak in FFT spectrum of the signal S(f), as it is shown in Figure 6.
- Amplitude of the signal with the corresponding mean, standard deviation (std) and interquartile range (iqr). The amplitude of the signal is calculated as it is shown in Figure 7, according to the following equations:
  (1) $$A_{i}^{-}=|p_{i}^{+}-p_{i}^{-}|;A_{i}^{+}=\left|p_{i+1}^{-}-p_{i}^{+}\right|.$$
- Signal regularity with the corresponding mean, standard deviation and interquartile range. This metric is used to describe the regularity of signal period $\Delta T_{i}$, as it is shown in Figure 7 and calculated as follows:
  (2) $$\Delta T_{i}=t_{i+1}^{+}-t_{i}^{+}.$$
- Inter-cycle variation of frequency (ICVF) of the signal with the corresponding mean, standard deviation and interquartile range. It is calculated similarly to the signal regularity and it is related to signal regularity through $f_{i}=1/\Delta T_{i}$, as follows:
  (3) $$\Delta f_{i}=f_{i+1}-f_{i}.$$
- Signal power concentration ratio (SPCR), which represents the ratio of signal power in range ($f_{c}-0.5$ Hz, $f_{c}+0.5$ Hz) to the signal power in range ($f_{c}-2$ Hz, $f_{c}+2$ Hz), as follows:
  (4) $$P_{R}=\frac{\int_{f_{c}-0.5Hz}^{f_{c}+0.5Hz}S\left(f\right)df}{\int_{f_{c}-2Hz}^{f_{c}+2Hz}S\left(f\right)df}.$$

The classification accuracy of aforementioned features in the differential diagnosis of patients belonging to A1 vs. B1, as well as A2 vs. B2 groups, is assessed by means of receiver operating characteristics (ROC) analysis.

## 3. Results and Discussion

In this section, we present the results of the analysis for signals acquired by a gyroscope for the group of patients described in Section 2.1.

### 3.1. Cervical Dystonia with and without Head Tremor (A1 vs. B1)

In Table 3, we present the mean and standard deviation of the features calculated from triaxial gyroscope signal measurements. The results are presented for patients from groups A1 and B1, along with the corresponding *p*-values. As we can see, there exists a significant difference between two groups (*p* < 0.05) for almost all the features, except Regularity std, Regularity iqr and ICVF mean. In the sequel, we will focus on the feature that shows the optimal performance in terms of discriminating between A1 and B1 groups of patients.

**Figure 8:**
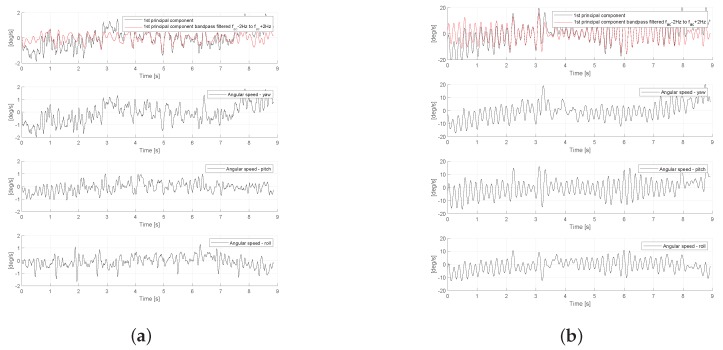
Gyroscope signal measured at the head of a patient with cervical dystonia. (**a**) Patient-1, without head tremor (from A1 group); (**b**) Patient-28, with head tremor (from B1 group).

In Figure 8, we present the measurements of triaxial gyroscope for two representative patients with cervical dystonia (Patient-1 from A1 group without clinically observable tremor and Patient-28 from B1 group, where tremor can be observed clinically). As can be seen in the figures, the gyroscope signal for the patient from group A1 has significantly smaller amplitude than the signal for patient from group B1 (with max amplitude on the order of ∼2 compared to ∼20 degrees/second). In addition, by simply observing the signals, we can see that the measured signal for the patient with tremor seems to be more regular. This can be explained by the fact that the signals measured in the head of patients without tremor can actually be attributed to small movements that are not periodical, which can be even observed in healthy subjects as well. Nevertheless, the patient with tremor presents a periodic movement type, whose amplitude may vary in time, as can be seen in Figure 8b. This behavior will be more clear later, when we present the frequency analysis of the signals.

**Figure 9:**
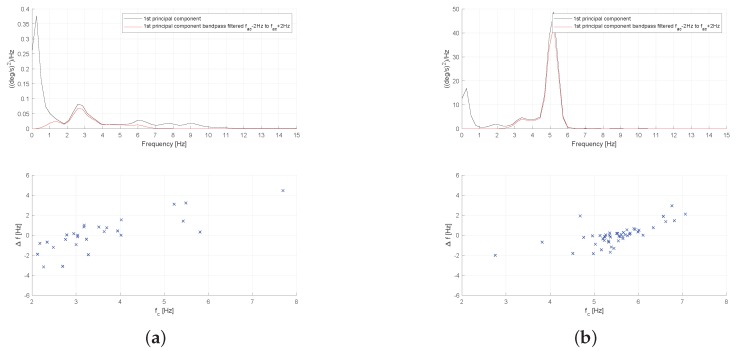
Power spectral density and Inter-cycle variation of gyroscope signal frequency measured at the head of a patient with cervical dystonia. (**a**) Patient-1, without head tremor (from A1 group); (**b**) Patient-28, with head tremor (from B1 group).

In Figure 9, we present the power spectral density (PSD) and inter cycle variation of signal frequency for two aforementioned patients (Patient-1 from A1 group and Patient-28 from B1 group). As we can see, the PSD of the gyroscope signal (actually the 1st principal component of the triaxial signals vector) shows a prominent peak at frequency 5.21 Hz, and, at the same time, its inter cycle variation of frequency is smaller as can be seen at the bottom Figure 9b, where we observe a higher concentration of points around the peak frequency. This behavior can be further confirmed by looking at the mean values of ICVF std and ICVF iqr values in Table 3, which are significantly higher for the group of patients without tremor that belong to group A1.

**Figure 10:**
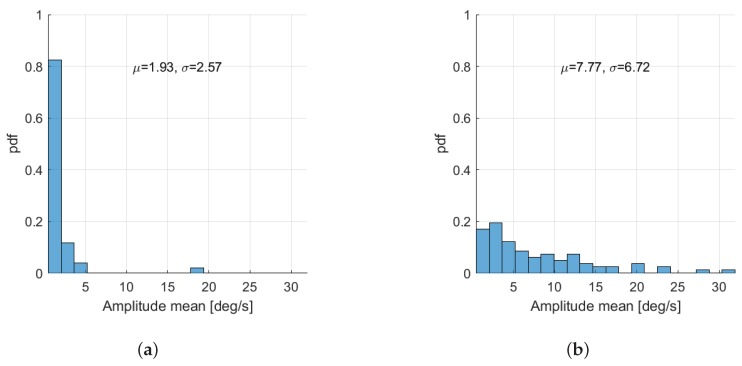
Distribution of the mean signal amplitude across the patients in A1 and B1 groups. (**a**) A1 group—patients without head tremor; (**b**) B1 group—patients with head tremor.

Next, in Figure 10, we present the distributions of the mean amplitude feature for A1 and B1 groups of patients, where the mean feature is calculated for each patient as the mean of amplitude defined in Equation (1). As we can see from Figure 10a,b, the mean of gyroscope signal amplitude for patients from the A1 group is significantly smaller than the mean amplitude for patients from the B1 group.

**Figure 11:**
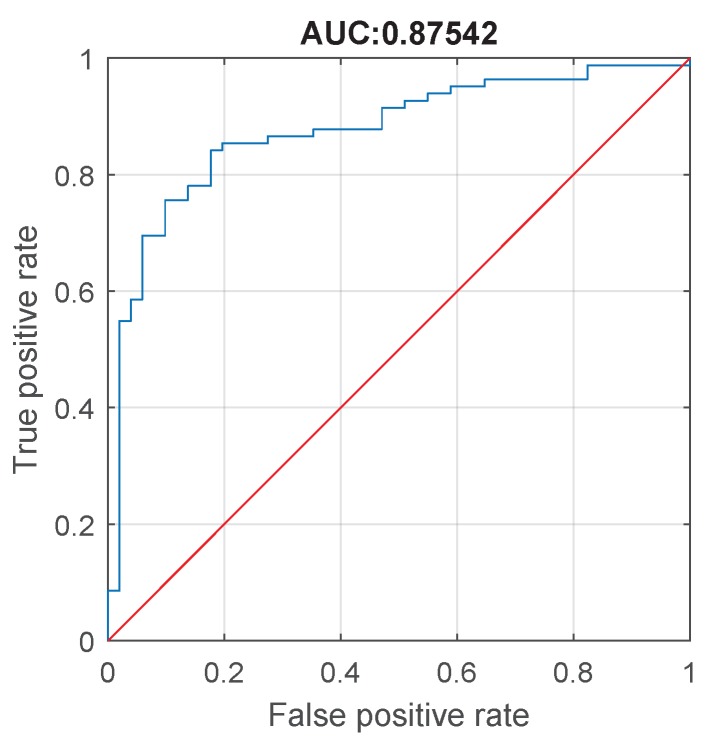
Receiver operating characteristic (ROC) for mean signal amplitude feature differentiating patients with cervical dystonia with and without tremor. Area under curve (AUC) is 0.87542 (95% confidence interval 0.79928–0.924).

The classification accuracy of the mean amplitude feature in the differential diagnosis of patients with cervical dystonia with and without tremor is assessed by means of receiver operating characteristics analysis that is presented in Figure 11. In this test, we consider as negative class group A1, and as the positive class group B1. The threshold for differentiation of Cervical dystonia tremor from essential tremor is identified by selecting the cutoff value that maximizes the distance between sensitivity (true positive rate) and 1-specificity (false positive rate). In particular, and having in mind that the specific threshold is dependent on the actual measured data, the optimal point is the one where the specificity = 0.8039 while sensitivity = 0.8537, which corresponds to the threshold equal to 2.0627 deg/s.

### 3.2. Essential Head Tremor vs. Cervical Dystonia with Head Tremor (A2 vs. B2)

In the sequel, we focus on the test involving A2 and B2 patient groups. In Table 4, we present the calculated features for the aforementioned patient groups, and present the corresponding mean, standard deviation and *p*-values. We find that, for some of the features, there is no significant difference between two groups. In line with the results presented in [19], we see that the mean frequency is not significantly different between groups A2 and B2. In addition, the amplitude mean is significantly higher in patients with essential tremor (A2) than in those having cervical dystonia. Finally, by considering the tremor irregularity, which can be measured by using ICVF iqr feature (similarly to Tremor Stability Index proposed in [18]), it can be seen that its value is significantly higher (30% more) in patients with cervical dystonia. This finding is in accordance with the results presented in [19]. Nevertheless, the authors in [19] consider limb tremor measured by using accelerometer sensor, in contrast to head tremor measured by using gyroscope considered in this paper. In addition, unlike [19], in this paper, we also consider spectral features of the tremor signal—SPCR, which will be presented in the sequel.

**Figure 12:**
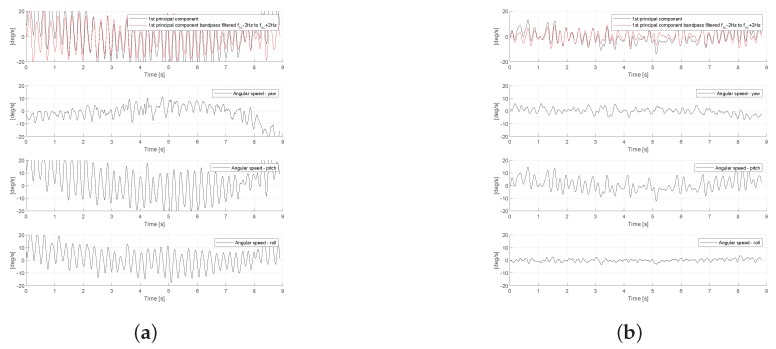
Gyroscope signal measured at the head of patients with essential tremor and cervical dystonia. (**a**) Patient-107, with essential tremor (from A2 group); (**b**) Patient-48, with cervical dystonia (from B2 group).

Next, in Figure 12, we present the measurements of triaxial gyroscope for two representative patients belonging to the groups with essential tremor (A2) and cervical dystonia (B2). As we can see, and in line with results presented in Table 4, the amplitude of the gyroscope signal (actually the first principal components of triaxial signal vector) is smaller for Patient-48 with cervical dystonia.

**Figure 13:**
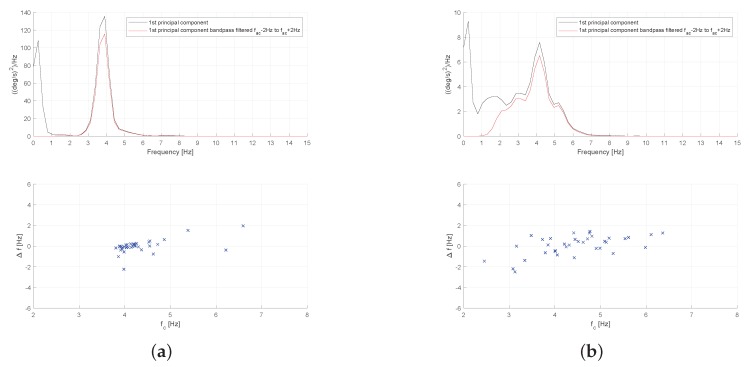
Power spectral density and Inter-cycle variation of gyroscope signal frequency measured at the head of patients with essential tremor and cervical dystonia. (**a**) Patient-107, with essential tremor (from A2 group); (**b**) Patient-48, with cervical dystonia (from B2 group).

By considering the power spectral density presented in Figure 13, we can see that the power is more concentrated around the peak frequency for the patient with essential tremor, suggesting a more regular signal. This extent can be further validated by looking at the bottom figures in Figure 13, where we can see that the inter cycle variation of frequency is smaller for Patient-107 (more concentrated points) which was diagnosed with essential tremor. Again, this is in line with the results presented in Table 4, in particular with the mean of ICVF iqr feature, which is used as a measure of frequency variability in time.

**Figure 14:**
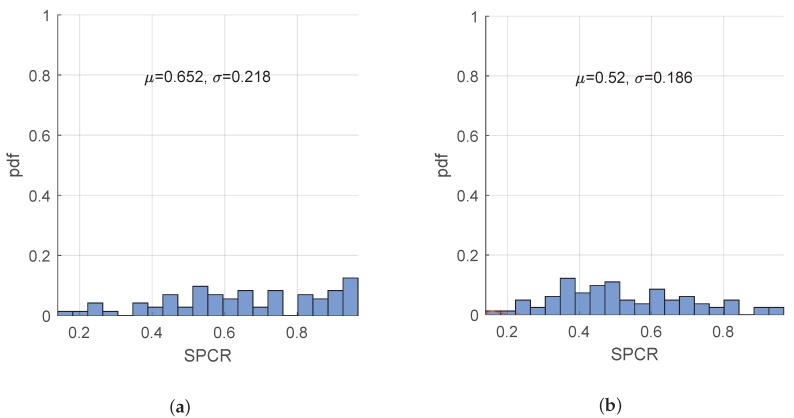
Distribution of the Signal Power Concentration Ratio (SPCR) across the patients in A2 and B2 groups. (**a**) A2 group—patients with essential tremor; (**b**) B2 group—patients with cervical dystonia.

Next, we consider SPCR as a potential measure to differentiate between groups A2 and B2, as it is shown in Figure 14. As expected, SPCR is distributed more towards 1 (μ=0.652), for patients in group A2 (essential tremor), which are more regular and tend to have most of their power distributed around the peak frequency. As for head tremor associated with cervical dystonia, we see that SPCR is distributed more towards lower values (μ=0.52), suggesting less concentrated power around the tremor peak frequency.

**Figure 15:**
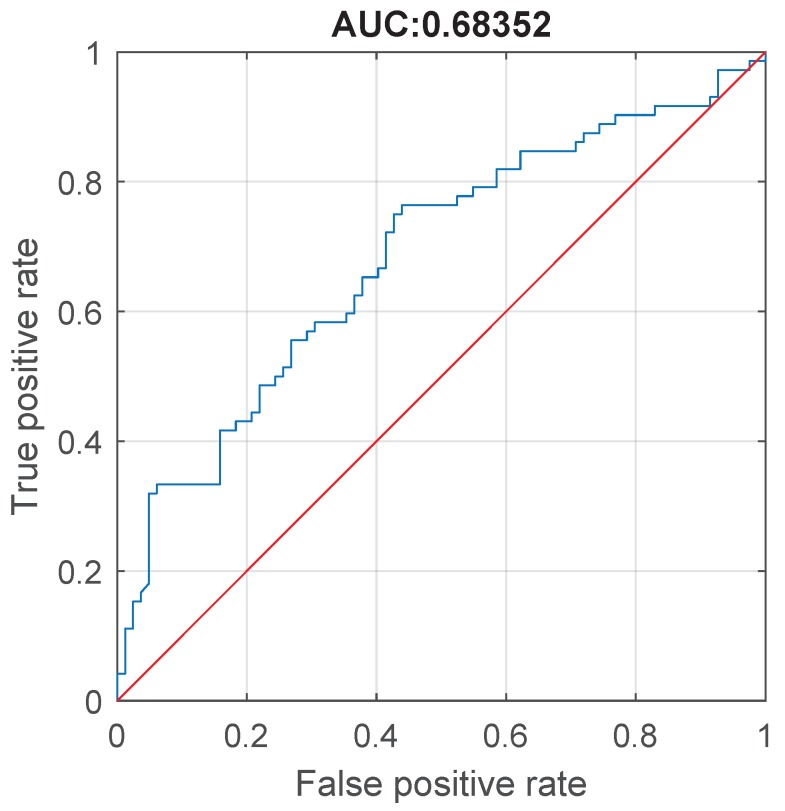
Receiver operating characteristic (ROC) for SPCR differentiating patients with essential tremor (A2) and cervical dystonia with head tremor (B2). Area under curve (AUC) is 0.68352 (95% confidence interval 0.58816–0.76259)

Again, we estimate the classification accuracy of SPCR feature in the differential diagnosis of patients with essential tremor and cervical dystonia with head tremor. It is assessed by means of receiver operating characteristics analysis, which is presented in Figure 15. In this test, we consider as positive class A2, and the negative class B2. Again, the threshold for differentiation of essential tremor from cervical dystonia is identified by selecting the cutoff value which maximizes the distance between sensitivity and 1-specificity. In particular, the optimal point is the one where the sensitivity = 0.75 while specificity = 0.5732, which corresponds to threshold 0.5202.

As a possible extension of this work, in the future, the authors will consider the analysis of cervical dystonia with head tremor in the case where the patient turns her head in the direction of dystonic position and in the opposite one. The aim of this future investigation will be to identify the characteristics of tremor in different positions and the effect of medication on the tremor itself.

## 4. Conclusions

In this paper, we proposed an architecture for remote health care within broader Ambient Assisted Living concept. In particular, we focused on the quantitative assessment of head tremor in patients with essential tremor and cervical dystonia by using inertial sensors. The specific aim was to test new tools for differentiating between these two movement disorders, based on a number of temporal and spectral features, which were calculated out of measured gyroscope signals. In particular, the mean signal amplitude feature was shown to have good differentiation capability for patients with cervical dystonia with (μ=7.77deg/s,σ=6.72deg/s) and without (μ=1.93deg/s,σ=2.57deg/s) head tremor, resulting in sensitivity = 0.8537 and specificity = 0.8039. Furthermore, for the test case involving patients with essential tremor and cervical dystonia with head tremor, we see that the mean frequency is not significantly different between two groups. In addition, the amplitude mean is shown to be significantly higher in patients with essential tremor than in those having cervical dystonia. In addition, by considering the ICVF iqr feature, it can be observed that its value is significantly higher (30% more) in patients with cervical dystonia than in essential tremor. Finally, we proposed the SPCR feature, which stands for the signal power concentration around peak frequency, that resulted in sensitivity = 0.75 and specificity = 0.5732 on the measured data set. The aforementioned results confirm that a wearable gyroscope sensing device can be used for monitoring of tremor in patients with movement disorders and for differentiation between essential tremor and cervical dystonia.

## Figures and Tables

**Table 1:** Clinical and demographic characteristics of the patients belonging to groups A1 and B1 (statistically significant *p*-values (*p* < 0.05) are highlighted with bold font).

Characteristic	Group A1	Group B1	*p*-Value
Number of patients	75	96	
Age *	49.9 ± 11.60	55.9 ± 12.29	**0.01**
Sex (M/F) ${}^{\#}$	28/47 (36.33%/62.67%)	31/65 (32.3%/67.7%)	0.41
Dominant hand (R/L) ${}^{\#}$	73/2 (97.33%/2.67%)	92/4 (95.83%/4.17%)	0.82
Age at disease onset *	41.3 ± 11.27	42.9 ± 13.05	**0.04**
Disease duration *	8.8 ± 6.01	12.7 ± 10.08	**0.007**

* mean ± standard deviation (μ±σ), ${}^{\#}$ numbers (percentage).

**Table 2:** Clinical and demographic characteristics of the patients belonging to groups A2 and B2 (statistically significant *p*-values (*p* < 0.05) are highlighted with bold font).

Characteristic	Group A2	Group B2	*p*-Value
Number of patients	78	96	
Age *	62.26 ± 13.01	55.9 ± 12.29	**0.004**
Sex (M/F) ${}^{\#}$	18/60 (23.1%/76.9%)	31/65 (32.3%/67.7%)	0.18
Dominant hand (R/L) ${}^{\#}$	77/1 (98.7%/1.3%)	92/4 (95.83%/4.17%)	0.26
Age at disease onset *	50.81 ± 15.82	42.9 ± 13.05	**0.001**
Disease duration *	12.09 ± 11.12	12.7 ± 10.08	1.00

* mean ± standard deviation (μ±σ), ${}^{\#}$ numbers (percentage).

**Table 3:** Mean and standard deviation of different features for patients in A1 and B1 groups (ICVF—Inter-cycle variation of frequency; SPCR—Signal power concentration ratio; mean—average value; std—standard deviation; iqr—interquartile range). Statistically significant *p*-values (*p* < 0.05) are highlighted with bold font.

Feature	Group A1 μ±σ	Group B1 μ±σ	*p*-Value
Peak frequency	4.92 ± 1.62	4.37 ± 1.22	**0.0408**
Amplitude mean (deg/s)	1.93 ± 2.57	7.77 ± 6.72	**1.2434 × 10${}^{-10}$**
Amplitude std (deg/s)	1.19 ± 0.969	5.26 ± 5.27	**1.0782 × 10${}^{-9}$**
Amplitude iqr (deg/s)	1.93 ± 2.57	7.77 ± 6.72	**1.2434 × 10${}^{-10}$**
Regularity mean (s)	0.192 ± 0.0428	0.213 ± 0.0455	**0.0067156**
Regularity std (s)	0.0529 ± 0.0241	0.0533 ± 0.0237	0.93321
Regularity iqr (s)	0.0681 ± 0.04	0.0643 ± 0.0381	0.58669
ICVF mean (Hz)	0.00649 ± 0.0702	−0.00285 ± 0.0696	0.45574
ICVF std (Hz)	2.68 ± 1.25	1.97 ± 1.03	**0.0011396**
ICVF iqr (Hz)	2.53 ± 0.953	2.07 ± 0.919	**0.0072191**
SPCR	45.9 ± 15.4	52 ± 18.6	**0.043076**

**Table 4:** Mean and standard deviation of different features for patients in A2 and B2 groups (ICVF—Inter-cycle variation of frequency; SPCR—Signal power concentration ratio; mean—average value; std—standard deviation; iqr—interquartile range). Statistically significant *p*-values (*p* < 0.05) are highlighted with bold font.

Feature	Group A2 μ±σ	Group B2 μ±σ	*p*-Value
Peak frequency	4.25 ± 0.883	4.37 ± 1.22	0.48278
Amplitude mean (deg/s)	12.3 ± 15.3	7.77 ± 6.72	**0.024072**
Amplitude std (deg/s)	5.83 ± 8.88	5.26 ± 5.27	0.63558
Amplitude iqr (deg/s)	12.3 ± 15.3	7.77 ± 6.72	**0.024072**
Regularity mean (s)	0.225 ± 0.0371	0.213 ± 0.0455	0.090724
Regularity std (s)	0.0446 ± 0.0224	0.0533 ± 0.0237	**0.021083**
Regularity iqr (s)	0.0544 ± 0.0356	0.0643 ± 0.0381	0.10118
ICVF mean (Hz)	−0.005 ± 0.0433	−0.00285 ± 0.0696	0.8165
ICVF std (Hz)	1.61 ± 1.18	1.97 ± 1.03	**0.041874**
ICVF iqr (Hz)	1.56 ± 1	2.07 ± 0.919	**0.001199**
SPCR	62.8 ± 21	49.3 ± 20.2	**7.9995 × 10${}^{-5}$**
